# Louisville Medical Institute

**Published:** 1837

**Authors:** 


### Art. IV.—Louisville Medical Institute.

Professors Caldwell, Cooke and Yandell, late incumbents of the Transylvania School, have been appointed to corresponding Chairs in the institution now forming in Louisville, under the title prefixed to this article. Dr. Henry Miller, a talented and respectable physician of that city, has been selected for the Obstetrical Chair. We understand that all these gentlemen have accepted.

From Professor Caldwell we learn, that the City Council of Louisville have made a munificent appropriation for the endowment of the Institute; and that he proposes to devote the remainder of his active life to its service. D.

